# Antioxidants as Renoprotective Agents for Ischemia during Partial Nephrectomy

**DOI:** 10.1155/2019/8575398

**Published:** 2019-02-07

**Authors:** Gabriela F. Buys-Gonçalves, Leonardo A. S. Abreu, Bianca M. Gregorio, Francisco J. B. Sampaio, Marco A. Pereira-Sampaio, Diogo B. de Souza

**Affiliations:** ^1^Urogenital Research Unit, Rio de Janeiro State University, Rio de Janeiro, RJ, Brazil; ^2^Faculty of Medicine, Estacio de Sá University, Rio de Janeiro, RJ, Brazil; ^3^Department of Morphology, Fluminense Federal University, Niteroi, RJ, Brazil

## Abstract

Small renal masses have been diagnosed increasingly in recent decades, allowing surgical treatment by partial nephrectomy. This treatment option is associated with better renal function preservation, in comparison with radical nephrectomy. However, for obtaining a bloodless field during surgery, occlusion of renal artery and veins is often required, which results in transitory ischemia. The renal ischemia-reperfusion injury is associated with increased reactive oxygen species production leading to renal tissue damage. Thus, the use of antioxidants has been advocated in the partial nephrectomy perioperative period. Several antioxidants were investigated in regard to renal ischemia-reperfusion injury. The present manuscript aims to present the literature on the most commonly studied antioxidants used during partial nephrectomy. The results of experimental and clinical studies using antioxidants during partial nephrectomy are reported. Further, alimentary sources of some antioxidants are presented, stimulating future studies focusing on perioperative antioxidant-rich diets.

## 1. Introduction

Renal cell cancer (RCC) arises mainly from the renal parenchyma and accounts for over 90% of kidney cancers. Incidence rates of RCC vary greatly worldwide, from 1.2 cases/100,000 in females from South Korea to 15.3/100,00 in males from Czech Republic [1]. In the United States the incidence of RCC rose consistently over the past three decades specially among early stage tumors [2]. Risk factors related to RCC include cigarette smoking, obesity, and hypertension. Physical activity and diets rich in antioxidants are inversely related to RCC. A status of increased reactive oxygen species (ROS) production and lipid peroxidation has been implicated in RCC carcinogenesis [3]. In favor of this hypothesis, several studies have evidenced a protective mechanism of antioxidants against RCC [4, 5].

As small renal masses are diagnosed more frequently, the incidence of nephron-sparing procedures has also increased [6]. Partial nephrectomy (PN) is the preferred treatment option for localized renal tumors according to most urological associations achieving oncological outcomes comparable to radical nephrectomy [7, 8]. In order to achieve a bloodless field during surgery, occlusion of renal artery and veins is often required.

Ischemia has been considered historically as a major factor in reducing renal function after PN [9]. Several measures to decrease the effects of ischemia have been used such as hypothermia and pharmacologic interventions [10, 11]. In this review, we assess some of the antioxidants that may be used for renal function preservation during PN.

## 2. Renal Ischemia-Reperfusion (I/R) Injury

The kidney is an organ supplied by end arteries, which means that the area irrigated by a given arterial branch will become ischemic if blood flow is interrupted by any reason. In contrast, the venous drainage has no segmental organization and anastomoses freely. During partial nephrectomy, ischemia may occur by both arterial and venous occlusion. However, the procedure may be carried by arterial occlusion only. Clinical and experimental studies have shown that when renal artery is clamped alone instead of both renal artery and vein, the injury is attenuated [12, 13]. Therefore, ischemic injury during partial nephrectomy may occur heterogeneously.

There are regions of the kidney that are more susceptible to ischemic injury. Epithelial cells located in the corticomedullary region are more susceptible to ischemia, since they have a greater oxidative activity and are located in an area with low oxygen reserve. The cells of the renal papilla reside in a naturally hypoxic environment and can withstand short periods of ischemia with anaerobic metabolism. The outer cortex is usually more resistant to ischemia because of its greater oxygen reserve [14]. Nevertheless, for very long periods of warm ischemia, all regions of the kidney are affected.

As previously mentioned interruption of arterial supply is often necessary during PN, and it gives rise to a chain of events that culminates in cell death if blood flow is not restored in a timely manner. Sutton and colleagues proposed a division of the clinical events of ischemic acute renal failure into 4 phases [15]: initiation, extension, maintenance, and recovery phase.

The initiation phase is characterized by cellular adenosine 5'-triphosphate (ATP) depletion with subsequent cellular electrolyte shifts, cellular swelling, and the induction of cellular stress responses. There are two biochemical events that must be emphasized as consequence of ATP depletion: rise in the concentration of hypoxanthine [16] and rise in both mitochondrial and cytosolic calcium levels [17].

**Figure 1:**
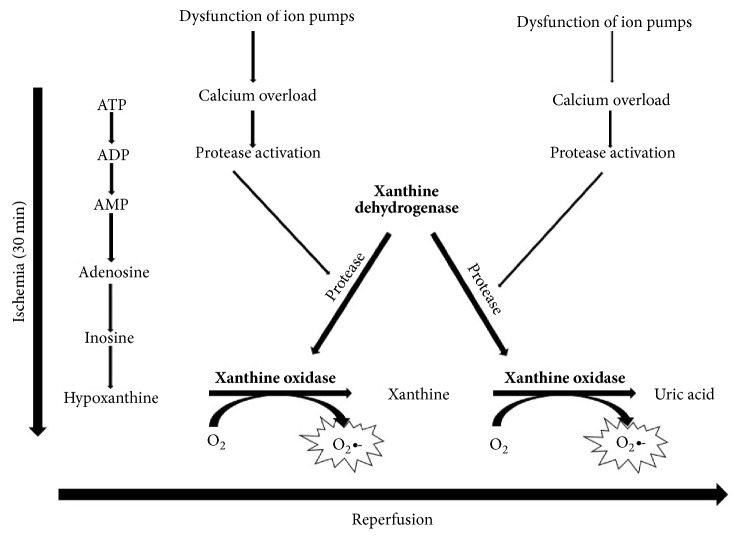
During ischemia, transmembrane ion gradients are dissipated, allowing cytosolic concentrations of calcium to rise, which in turn activates protease that irreversibly converts xanthine dehydrogenase into xanthine oxidase. At the same time, cellular ATP is catabolized to hypoxanthine, which accumulates. During the reperfusion, xanthine oxidase using readmitted oxygen and hypoxanthine generates superoxide and hydrogen peroxide. Scheme derived from Granger et al. (1986).

Hypoxanthine is a breakdown product of ATP metabolism and is, normally, oxidized by the enzyme xanthine dehydrogenase to uric acid. Hypoxanthine can also be oxidized by xanthine oxidase (XO), which is an isoform of xanthine dehydrogenase and transfers an electron to oxygen forming the free radical superoxide (O_2_•-). Conversion of xanthine dehydrogenase to oxidase may be influenced by several mechanisms during ischemia, and it takes about 30 minutes to occur in the kidney [18]. This may be a biochemical explanation for the safety limit of 25 minutes of warm ischemia observed in the clinical setting [19], although experimental studies have not supported this theory [20, 21]. Dysfunction of ATP-dependent membrane ion pumps with consequent rise in both mitochondrial and cytosolic calcium levels is another important event. Calcium overload leads to mitochondrial membrane dysfunction and irreversible damage (Figure 1).

The extension phase is characterized by the restoration of renal blood flow that starts various inflammatory events. Although blood flow is restored, reperfusion may lead to further injury as already shown in other organs [22]. Production of oxygen-derived free radicals is a major event that leads to tubular, vascular, and interstitial injury [23]. The segment S3 of proximal tubules is particularly susceptible to I/R injury [24]. Decreased renal function may ensue by backleak of the glomerular ultrafiltrate across the tubular epithelium. Also, tubular obstruction by cell debris may contribute to reduced glomerular filtration rate. Microvasculature injury is another important event during extension phase [15]. Endothelial and epithelial cells secrete inflammatory cytokines and express adhesion molecules that promote the activation of lymphocytes, margination, and diapedesis. Leukocyte infiltration, especially by neutrophils, leads to further production of cytokines and oxygen-derived free radicals, which in turn cause additional injury of to the epithelium and stroma.

During the maintenance phase, glomerular filtration rate stabilizes as cellular repair processes are initiated in order to maintain and reestablish organ integrity. The repair phase is characterized by cell proliferation and tissue repair with the recovery of subsequent kidney function and may last for weeks or months.

## 3. Free Radicals

**Figure 2:**
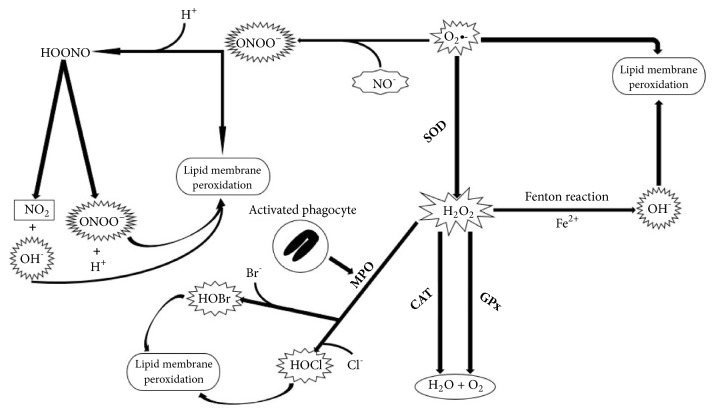
Role of superoxide anion in the generation of other reactive species. O_2_•- (superoxide); H_2_O_2_ (hydrogen peroxide); OH^−^ (hydroxyl); Fe^2+^ (iron); H_2_O (water); O_2_ (oxygen); NO- (nitric oxide); ONOO^−^ (peroxynitrite); H^+^ (hydrogen); HOONO (peroxynitrous acid); NO_2_ (nitrogendioxide); Br^−^ (bromide); Cl^−^ (chloride); HOBr(hypobromous acid); HOCl (hypochlorous acid); SOD (superoxide dismutase); CAT (catalase); GPx (glutathione peroxidase); MPO (myeloperoxidase).

A free radical, also known as reactive oxygen species (ROS), has one or more unpaired electron and so is chemically highly reactive. ROS are commonly related to aerobic metabolism and birth immunity. On the other hand, they may have also important signaling and/or regulatory function in living organisms [25]. In the renal parenchyma, free radicals are produced by components of connective tissue, epithelial and muscular, like fibroblasts, endothelial cells, vascular smooth muscle cells, mesangial cells, tubular cells, and podocytes cells [26]. Ordinary ROS implicated in ischemic kidney injury are as follows: superoxide (O_2_•-), hydrogen peroxide (H_2_O_2_), hydroxyl (OH^−^), nitric oxide (NO), and the peroxynitrite anion. O_2_•- is a byproduct of normal cellular metabolism and is generated as oxygen accepts a single electron and can inactivate specific enzymes but, more meaningfully, it may lead to production of two other highly reactive species: H_2_O_2_ and OH^−^. The dismutation of O_2_•- by superoxide dismutase (SOD) generates H_2_O_2_, which in turn can inactivate DNA [27], impair ATP synthesis, and inhibit glycolysis [28] leading to elevation in intracellular calcium, disruption of the cytoskeleton, blebbing of the plasma membrane, and finally cell death. The deleterious effects of superoxide, however, can be offset by catalase (CAT) and glutathione peroxidase (GPx) (Figure 2).

The interaction of O_2_•- and H_2_O_2_ catalyzed by molecular iron (Fenton reaction) originates OH^−^ [29]. The OH^−^ radical is extremely reactive and is supposed to be in charge for most of the cellular damage that occurs from ROS [30] (Figure 2). Fortunately, there are several scavengers that stabilize OH^−^ effect, comprehending tryptophan, histidine, ascorbate, and alpha-tocopherol [31].

NO and O_2_•- anions can react to compose peroxynitrite, which may lead to oxidation of a wide chain of biological targets including amino acids such as cysteine, methionine, tyrosine and tryptophan, nucleic bases, and antioxidants [32]. Peroxynitrite reacts with aim molecules through two potential pathways: it may react directly with a target molecule or it can dissociate in peroxynitrous acid and homolyze to form nitrogen dioxide and OH^−^ radicals, which in turn react with the aim molecule (Figure 2). S-methylisothiourea, an iNOS inhibitor, and mercaptoethylguanidine, a scavenger of peroxynitrite, have shown protective effect on renal I/R injury [33].

One of the most abundant enzymes liberated on neutrophil activation, myeloperoxidase (MPO), is a 140-kDa heme protein released by activated phagocytes in the course of inflammatory process. It catalyzes the reaction of H_2_O_2_ with physiological convergences of chloride and bromide anions to produce hypochlorous acid (HOCl) and hypobromous acid (HOBr), respectively [34], which are oxidants and electrophiles that react easily with biological components such as proteins, lipids, and DNA as well [35–37] (Figure 2). Thus, cellular damage resulting from excessive or misplaced production of hypohalous acids has been implicated in reperfusion injury [38]. A research used MPO-deficient mice (Mpo -/-) compared to controls after kidney I/R found 24 hours later significant reduction in renal function decrease in Mpo -/- mice compared with I/R controls, as a reduced neutrophil influx [39]. Another study used the porcine kidney with gene deletion for MPO+ neutrophils and found a 91% decrease of apoptosis in nephrotic tubule cells and amelioration of renal function after I/R [40].

To evaluate the role played by ROS, indirect biomarkers are usually searched [39, 41], because it is difficult to perform straight assessment of such unstable reactive species [42]. The antioxidant activity is measured through enzymes as CAT, GPx, MPO, and SOD. Besides the enzymes, the lipid peroxidation may be evaluated through TBARS determination (directly related to the production of malondialdehyde (MDA)). SOD, CAT, GPx, and MPO are evaluated from tissues samples by immunohistochemistry and TBARS by Enzyme-Linked Immunosorbent Assay (ELISA). To systemic assessment, f2-isoprostanes (F2IP) from plasma can be useful to observe lipid peroxidation [43–45].

## 4. Antioxidants and Renal I/R Injury

Several antioxidants are investigated regarding I/R injury. Commonly studied antioxidants are listed in Table 1. A brief description of some of the most important antioxidants follows.

### 4.1. Naturally Occurring Enzymatic Antioxidants

**Catalase (CAT): **Enzyme which is present mainly in the peroxisomes of mammalian cells. If the concentration of H_2_O_2_ is high, CAT acts catalytically and removes H_2_O_2_ by forming H_2_O and O_2_ [46]. A study made use of this chemical reaction both to determine whether the inhibition of the H_2_O_2_ catalyzing enzyme would influence ischemic renal injury and to determine the rates of H_2_O_2_ formation after ischemia. Inhibition of CAT prior to ischemia led to an increasement of ischemic injury. The production of H_2_O_2_ occurs in both normal and ischemic kidneys even though intracellular sites and production rates are likely to be diverse. CAT is an essential protective enzyme, since its inhibition leads to exaggerated post-ischemic renal dysfunction [47]. Overexpression of CAT prevented apoptosis-inducing factor (AIF) translocation from mitochondria to the nucleus, reducing ROS charge after ischemia [48]. A research in rats concluded that CAT protein overexpression by adenoviral CAT gene (Adv-CAT) transfection improved I/R-induced injury in the kidney by reducing H_2_O_2_, serum urea, and glutathione s-transferase levels. During post-ischemic reperfusion, leftover ROS production from mitochondria begins apoptosis via the release of cytochrome c from it, which was restrained by Adv-CAT, accordingly, depressing I/R-enhanced autophagy-related proteins and apoptosis-mediated proteins expression. This technique does not impel nephrotoxicity and CD4+/CD8+-mediated immune response in the treated kidneys, which gives to this kind of treatment a greater translational aspect [49].

**Glutathione peroxidase (GPx): **This enzyme is one of the main endogenous antioxidant defenses that work in higher organisms and catalyze the reduction of H_2_O_2_ or organic hydroperoxides to H_2_O or analogous alcohols. A classic study in rats used the redox ratio of GPx to assess levels of peroxidation through the H_2_O_2_ present in the renal parenchyma to fix upon the severity of the ischemic injury and obtained significant results [50]. There is a research which used the murine model with human GPx1 and GPxP gene overexpression that showed up more resistant to damage caused by I/R in the kidneys by reduction of mortality and serum urea and creatinine levels, tubular necrosis, apoptosis, oxidative stress and lipid peroxidation, MDA, MPO activity, expression of mRNA, and inflammatory cytokines six hours after reperfusion. The most relevant of cytokines was MIP-2, related to greatest migration of leukocytes which also had a lower activity in the transgenic groups GPx1 and GPxP. There was also a decrease in the activity of NF-kB, a transcription factor acknowledged to be responsible for the activation of numerous genes mediating the inflammatory process in general as well as during I/R in groups GPx1 and GPxP, so GPx seems to be involved in the inhibition of the activation of the MIP-2 promoter gene by NF-kB [51]. Zemlyak and colleagues tested the overexpression of GPx in cells after ischemia and it prevented apoptosis-inducing factor (AIF) translocation from mitochondria to the nucleus. This could reflect mainly non-specific scavenging ROS [48]. Another study shows that the use of glutamine amino acid supplementation, a precursor of GPx, prior to renal I/R elevates this powerful endogenous antioxidant in rats, which again proved to be efficient as an endogenous scavenger that preserved renal function [52].

**Superoxide dismutase (SOD)**: It is possible that SOD is an enzyme with real anti-aging consequences and can act positively over all the degenerative processes. The preventive effects of intravenously exogenous SOD on acute renal failure were investigated in the kidneys of rats exposed to warm ischemia. In an experiment, SOD was given just before primary ischemia and in the early recirculation phase. It was found to ameliorate the red cell aggregation in the renal medulla in the inner stripe of the outer zone. The volume of trapped red cells decreased in treated animals, thus allowing better restoration of medullary blood flow. SOD also restored the capillary macromolecular permeability as shown by standardization of plasma to lymph transport of proteins. Ischemically damaged but untreated kidneys had the tubules obstructed and that the proximal tubular pressure rose to such a level that the net driving force for filtration approached zero, explaining the marked decrease in glomerular filtration rate (GFR) from a normal value [53]. Dogs were used as models for evaluation whether the administration of SOD can alleviate I/R renal damage and whether there is a relationship between oxygen free radicals and thromboxane (Tx). Blood samples were drawn from the renal vein before ischemia and after reperfusion to assess serum levels of thromboxane B2 (TxB2). All untreated dogs died within seven days of renal failure and the treated ones demonstrated transient renal failure, with a significant difference being found between groups. A significant difference in TxB2 levels was found in the untreated dogs before and after ischemia and between the two groups after reperfusion. Animals that were treated with exogenous SOD after the ischemic event has occurred but before reperfusion showed a favorable clinical course in terms of survival and renal function [54]. A study in rats showed that the renal protective effect of free SOD on warm ischemic-reperfusion injury is conditional on the time of administration, being further effective when given prior to reperfusion. On the other hand, the renal protective effect of liposomal SOD did not depend on the time of administration since efficacy was similar when given before reperfusion or ischemia. It was concluded that liposomal SOD shows a higher renal protective effect in warm ischemia than free SOD [55]. Zemlyak and colleagues tested the overexpression of CuZnSOD (extracellular and cytosolic SOD) or MnSOD (mitochondrial SOD) in cells after ischemia and both prevented AIF translocation from mitochondria to the nucleus which could reflect broadly non-specific protection due to reducing ROS [48].

### 4.2. Naturally Occurring Exogenous Antioxidants

**Curcumin: **Curcumin is a frequently studied phenolic compound. Extracted from* Curcuma longa*, curcumin is a bifunctional antioxidant, often added to mustard, condiments, and sauces and it exerts antioxidant activity in a direct and an indirect way by scavenging reactive oxygen species or inducing an antioxidant response, respectively [56]. In a study, rat kidneys with I/R injury were analyzed for serum and tissue NO, protein carbonyl, MDA, SOD, and GPx levels. Histopathological examinations were also performed. Reduction of serum GPx was significantly improved by curcumin, but SOD enzyme activity was not altered. Treatment with curcumin also resulted in significant reduction in serum and tissue MDA, NO, and protein carbonyl. In histological examination, the rats treated with curcumin had nearly normal morphology of the kidney [57]. Another research, also with rat kidneys, aimed to investigate the role of N-methyl-d-aspartate (NMDA) receptors in curcumin-mediated renoprotection against I/R injury. In separate groups, NMDA receptor agonists (glutamic acid and spermidine) were injected prior to curcumin treatment followed by renal I/R, and administration of curcumin resulted in significant protection against I/R injury in the sham group. However, glutamic acid and spermidine pretreatments prevented curcumin-mediated renoprotection allowing the conclusion that NMDA receptor antagonism significantly contributes towards curcumin-mediated protection against I/R injury in rats [58].

Ferulic acid belongs to the phenolic acid group commonly found in plant tissues [59] and is most commonly found in grains, spinach, parsley, grapes, rhubarb, and cereal seeds, being more easily absorbed and stays in the blood longer than any other phenolic acids [60]. The antioxidant mechanism of ferulic acid is based on raising inhibition and scavenging of ROS and on blocking enzymes that catalyze their production, such as MPO, and is also an enhancer of scavenger enzyme activity [61–64]. Antioxidant activity of ferulic acid is forming stable phenoxyl radicals by the reaction of the radical molecule with the stable antioxidant molecule and may also act as hydrogen donor. As a secondary antioxidant, ferulic acids and their related compounds can bind transition metals such as iron and copper, preventing the formation of toxic OH^−^ radicals [65]. A recent study in the rodent model of I/R showed that ferulic acid significantly attenuated kidney damage by decreasing levels of urea and creatinine, pathological structural changes, and tubular cells apoptosis, inhibited I/R-induced renal proinflammatory cytokines and neutrophils recruitment, and increased adenosine generation and CD39 and CD73 expression [66].

**Ligustrazine**: Ligustrazine is an alkaloid isolated from the rhizome of Chuanxiong (*Ligusticum chuanxiong *Hort), which is notorious by its antioxidant, anti-inflammatory, anti-fibrosis, and immunomodulative effects [67]. It is found in cocoa bean or soybean-based fermented foods, Chinese alcohols, and soybeans culture media of* Bacillus subtilis*, among others. The effects of ligustrazine on oxidative stress, neutrophils recruitment, proinflammatory mediators, and adhesion molecules caused by renal I/R injury were assayed in mice, and its pretreatment attenuated dramatically the injuries in kidneys caused by warm ischemia, reducing MPO activity and decreasing MDA level, while SOD activity increased, suggesting an effective reduction of oxidative stress. Moreover, ligustrazine also inhibited cell apoptosis, abrogated neutrophils recruitment, and suppressed the overexpression of tumor necrosis factor-alpha (TNF-*α*) [68].

**Quercetin**: Quercetin is one of the most potent scavengers of ROS in the family of polyphenolic compounds [69], found in fruits (citrus fruits, apples, grapes, dark cherries, and dark berries), vegetables (onions, parsley, and sage), tea, olive oil, and red wine. TBARS, protein carbonyl content, TNF-*α*, GSH levels, MPO, CAT, and SOD activities were determined in renal tissue in a study with renal I/R rat model. Its administration previously to I/R decreased the oxidation and inflammatory parameters (TBARS, TNF-*α* levels, MPO activity, and protein carbonyl content). Quercetin treatment significantly increased reduced glutathione (GSH) levels and activities of SOD and CAT when compared to the I/R group [70]. This substance made MDA levels significantly decrease after I/R in another research in rats and significantly increased glutathione level. In histological results, the number of apoptotic and endothelial nitric oxide synthase (eNOS) expression levels were significantly decreased in the quercetin treated group [71]. A third study determined the effects of quercetin on AMPK and autophagy signals in the kidneys of mice after I/R. Quercetin significantly increased the phosphorylation of AMPK and decreased the phosphorylation of the mammalian target of rapamycin (mTOR), one of the downstream targets of AMP-activated protein kinase (AMPK) [72].

**Resveratrol**: As quercetin, it is a bioflavonoid. Resveratrol is a polyphenolic compound found in grapes, berries, and peanuts. It possesses a variety of bioactivities, including antioxidant, anti-inflammatory, and renal protective effects [73]. Previous studies have shown that resveratrol can directly scavenge reactive oxygen species (ROS) [74]. In addition to scavenging ROS, exogenously administered resveratrol modulates the expression and activity of antioxidant enzymes, such as SOD, GPx, and CAT, either through transcriptional regulation via nuclear factor E2-related factor 2 (Nrf2), activatorprotein (AP) 1, and forkhead box protein O (FOXO), or through enzymatic modifications [75]. A recent study demonstrated that resveratrol activates 2 homolog sirtuin 1 (SIRT1) that may regulate multiple cellular functions, including apoptosis, mitochondrial biogenesis, inflammation, glucose/lipid metabolism, autophagy, and adaptations to cellular stress, through the deacetylation of target proteins through the activation of AMPK [76].

**Vitamin C**: Ascorbic acid, also known as vitamin C, is found in fruits (citrus fruits, mango, and avocado) and vegetables (broccoli, cauliflower, peppers, and asparagus) being widely accepted as an anti-oxidant and is an essential nutrient required for various metabolic reactions. The active part of ascorbic acid is ascorbate ion that acts as an electron-donating entity and is involved in biosynthesis of steroids, collagen, and peptide hormones. Vitamin C is a redox catalyst which itself gets reduced and neutralizes ROS such as H_2_O_2_. The donation of one electron by ascorbate results in semi-dehydroascorbate radical that is reduced by glutathione and NADPH-dependent enzymes [77]. Moreover, ascorbic acid increases the activity of endogenous antioxidant defense including SOD, GSH, and CAT in rats whose had induced I/R injury [78]. The administration of ascorbic acid in a study with rats showed significant increase in NO level along with decrease in oxidative stress. NO level may be due to scavenging of oxidative species such as O_2_•-. The same research proves that NO and soluble guanylyl cyclase pathway finds its definite involvement in ascorbic acid mediated protection against I/R injury [79]. Vitamin C supplementation preserved kidney morphology and renal function following I/R injury and decreased resistance index of renal artery, ameliorating oxidative stress secondary to I/R in a recent research performed in rat model [80].

**Vitamin E**: This liposoluble vitamin acts as a scavenger, protective against free oxygen species occurring during the reperfusion phase after renal ischemia, prevents lipid peroxidation, and acts against the effects of oxidative stress. Various foods provide vitamin E such as meat (egg and fish), nuts, seeds, vegetable oils, and fruits (tomato and avocado) [81]. A study in rats showed that the administration of vitamin E before and after renal I/R normalized the parameters of GSH, y-glutamyl-transpeptidase, and TBARS. It also improved the survival rate in adult rats up to 100% [82]. Another research used young adult, middle-aged, and aged rats to prove that a diet with vitamin E supplementation is essential for protecting aging kidneys against ischemic acute renal failure. The older animals with vitamin E deficiency had aggravated acute damage caused by I/R and in the absence of vitamin E and MDA levels increased with age [83]. Histopathologic examination of the rabbit kidney submitted to I/R pretreated with vitamin E showed normal histologic appearance with no sign of tubular necrosis and the nontreated showed moderate to severe ischemic changes. So, the intensity of I/R injury is less extensive in rabbits that received intravenous pretreatment with vitamin E before surgery [84].

### 4.3. Other Studied (and Utilized) Antioxidants in Renal I/R Injury

**Allopurinol**: Allopurinol is an inhibitor of XO which inhibits the conversion of hypoxanthine into xanthine and then uric acid as the final product of purine catabolism. During hypoxanthine conversion, O_2_•- and another ROS are generated. The inhibitory effect of allopurinol blocks the chain of events of the oxidative stress in an early stage. Recently, Prieto-Moure and colleagues [85] reviewed several studies in which the renoprotective effect of allopurinol was assessed. In most studies, allopurinol was administered prior to the ischemia period and the effective dose was 100 mg/kg. Clinical studies showed that allopurinol can be safely used in patients with chronic kidney disease (CKD) [103]. It also may delay progression of CKD although confirmation is still needed. Allopurinol is also used in organ transplantation. One of the most used preservation solutions developed by the University of Winscosin has allopurinol on its formulation [104]. Future studies to address the role of allopurinol in the context of partial nephrectomy are needed.

**Mannitol**: Mannitol, an osmotic diuretic, is used in the clinical perioperative setting in the belief that it exerts renoprotective properties. Bragadottir and colleagues evidenced renal vasodilation and redistributes systemic blood flow to the kidneys [105]. In the rabbit model, administration of mannitol before ischemia and before reperfusion reduced ROS production significantly. Glomerular function measured 48 h after reperfusion was significantly better after pretreatment mannitol [106]. A study using rats as a model showed that mannitol treatment significantly decreased the level of MDA, SOD, and MPO activity and increased GSH level (nonenzymatic antioxidant in the kidney tissues). Histological evaluation of kidneys demonstrates that mannitol significantly decreased tubular necrosis and inflammatory infiltration [81]. A recent study used the porcine model of I/R with positive results: kidneys subjected to ischemia displayed decreased weight, volume, and number of glomeruli in comparison to the sham operated and mannitol groups and concluded that using this antioxidant significantly reduces nephron loss during warm ischemia in this animal model [107].

**Nitric Oxide (NO)**: Endogen endothelial NO is an autacoid whose primary function is to decrease renal vascular resistance. There is a basal level of NO release, which acts to prevent excessive vasoconstriction in the kidneys, allowing a good excretion of sodium and water. It has been noticed that NO, this small molecule with multiple physiological functions, plays an important role in modulating tissue injury and renal blood flow in the healthy kidneys as well as several pathologic kidney conditions. The role of NO in I/R injury is controversial [108]. Exogenous NO has a beneficial effect in renal I/R injury, while endogenous NO does not appear to be an important contributor to renal I/R injury. The infiltration of neutrophils (migration) was decreased in a study in animals pretreated with the NO donor: Na-nitroprusside [96]. In vitro studies have shown that NO and peroxynitrite at high concentrations regulate XO activity [97]. A study utilized an exogenous Na-nitroprusside, to treat rats during reperfusion. The lipid peroxidation level was measured to determine oxidative damage, XO specific activity to evaluate O_2_•- production as well as renal GSH level, GPx, and SOD specific activities to determine the antioxidative capacity in renal tissue. It was examined whether exogenous NO has an in vivo inhibitory effect on XO activity in renal I/R injury and whether it has a protective effect on oxidative stress. GSH levels were lower in all treated kidneys compared to their control counterparts. The XO activity of ischemic kidneys of the group treated with Na-nitroprusside was lower than those of the other groups. Histological evaluation revealed that the median value of the damage grade of the Na-nitroprusside group was lower than that of the control group [98].

## 5. Antioxidants and Partial Nephrectomy

Several measures to decrease the impact of ischemia or even to avoid ischemia have been developed for the treatment of renal neoplasms. An experimental imaging study using in vivo murine model of renal ischemia made contribution showing usefulness of novel imaging technologies (electron paramagnetic resonance, EPRI) in measuring renal reducing activity and the evaluation of oxidative stress in post-ischemic renal disease [109]. In the surgical field, ablative procedures such as cryoablation and radiofrequency ablation are examples of procedures that avoid ischemia of the remaining parenchyma. Technical modifications of the partial nephrectomy procedure have also been used to reduce ischemia. “Zero ischemia” PN has been described by Gill and colleagues [110], although systemic hypotension is induced during tumor resection which may cause ischemia in a lesser degree. Partial nephrectomy assisted by focal radiofrequency is another example of nonischemic technique [98]. Even though occlusion of renal vessels during PN is necessary in several occasions, ablative procedures such as radiofrequency and cryoablation may also be used without ischemia. Nonetheless, PN remains the standard treatment of renal cell cancer in most cases.

Antioxidants have already been used in clinical practice to decrease renal I/R injury in transplant procedures. Clinical studies on the use of antioxidants during partial nephrectomy are scarce. The most frequently used renoprotective medication is mannitol [111], which is supposed to exert antioxidant effects [106, 112] as well as diuretic activity. Nonetheless, its beneficial effect on renal function is controversial. Two retrospective studies have shown no advantage from the administration of mannitol during partial nephrectomy [113, 114]. In both studies, however, a randomized controlled trial is suggested in order to clarify the role of mannitol during partial nephrectomy.

An ideal renoprotective medication for PN should be effective in preventing I/R injury as well as having low incidence of adverse events. Vasoactive drugs and medications that interfere with coagulation should be of concern because of the risk of bleeding in the intra- and postoperative period, for example. Also, medications should have a favorable posology and low cost.

In addition to the potential benefits of antioxidants in the acute setting of PN, their long-term use might also be investigated. The relation of lipid peroxidation to the pathogenesis of renal cell cancer as stated by Gago-Dominguez et al. [4] opens a great field of investigation in the preventive medicine. The effect of antioxidants on cancer recurrence may be considered for those already treated with curative intent.

## 6. Conclusions

Since the beginning of renal surgery, studies with medications to help preserve kidney function have been suggested. Nonetheless, little progress has been made in the context of PN. Antioxidants have the potential to improve the functional outcomes of partial nephrectomy, although both experimental and clinical data are still missing. Most studied antioxidant for application during partial nephrectomy can be found in dietary sources. Pre- and postoperative diets with nutraceuticals (antioxidant) aliments should be investigated for possible applications in partial nephrectomy perioperative nutrition.

## Figures and Tables

**Table 1:** Antioxidants commonly used in renal ischemia-reperfusion injury.

**Antioxidant**	**Mechanism of action**	**Improvement of I-R injury**	**Reference**
Allopurinol	Xanthine oxidase inhibitor	Yes	[85]

Amifostine	Increase in glutathione peroxidase	Yes	[86, 87]

Bilirrubin	Superoxide scavenger / peroxyl radical trapping antioxidant	Yes	[88, 89]

Catalase	Superoxide scanvenger	Yes	[46–49]

Ceruloplasmine	ROS scavenger / Fenton reaction inhibition	Yes	[90]

Coenzyme Q10	ROS scavenger / enhance antioxidants / quench perferryl radical	Yes	[91, 92]

Crocin	ROS scavenger	Yes	[93]

Curcumin	ROS scavenger / enhance antioxidants	Yes	[56–58]

Desferrioxamine	Iron-chelator / enhance antioxidants	Yes	[90, 94]

Edaravone	ROS scavenger	Yes	[95]

Ferulic acid	ROS scavenger/ enhance antioxidants/ Modulates MPO and other enzymes	Yes	[61–66]

Glutathione peroxidase	ROS scavenger / NF-*κ*B pathway inhibitor	Yes	[48, 50–52]

Ligustrazine	ROS scavenger	Yes	[67, 68]

Mannitol	ROS scavenger / enhance antioxidants	Yes	[81]

Nitric Oxide	Modulates xanthine oxidase activity / vasodilation	Yes	[96–99]

Quercetin	ROS scavenger	Yes	[69–72]

Resveratrol	ROS scavenger	Yes	[71–76]

Superoxide dismutase	Superoxide scavenger	Yes	[48, 53–55]

Vitamin C	ROS scavenger / enhance antioxidants	Yes	[77–79, 100–102]

Vitamin E	ROS scavenger / enhance antioxidants	Yes	[81–84]
